# Liver X Receptor exerts a protective effect against the oxidative stress in the peripheral nerve

**DOI:** 10.1038/s41598-018-20980-3

**Published:** 2018-02-06

**Authors:** Mehdi Hichor, Venkat Krishnan Sundaram, Stéphanie A. Eid, Ronza Abdel-Rassoul, Patrice X. Petit, Didier Borderie, Jean Bastin, Assaad A. Eid, Marin Manuel, Julien Grenier, Charbel Massaad

**Affiliations:** 1 Paris Descartes University, INSERM UMR-S 1124, Faculty of Basic and Biomedical Sciences, 45 rue des Saints-Pères, 75270 Paris Cedex 6, France; 2 American University of Beirut, Department of Anatomy, Cell Biology and Physiological Sciences, PO Box 11-0236, Riad El-Solh, 1107 2020, Beirut, Lebanon, Beirut, Lebanon; 3 Centre de Neurophysique, Physiologie et Pathologie, Université Paris Descartes, CNRS UMR 8119 Paris, France

## Abstract

Reactive oxygen species (ROS) modify proteins and lipids leading to deleterious outcomes. Thus, maintaining their homeostatic levels is vital. This study highlights the endogenous role of LXRs (LXRα and β) in the regulation of oxidative stress in peripheral nerves. We report that the genetic ablation of both LXR isoforms in mice (LXRdKO) provokes significant locomotor defects correlated with enhanced anion superoxide production, lipid oxidization and protein carbonylation in the sciatic nerves despite the activation of Nrf2-dependant antioxidant response. Interestingly, the reactive oxygen species scavenger N-acetylcysteine counteracts behavioral, electrophysical, ultrastructural and biochemical alterations in LXRdKO mice. Furthermore, Schwann cells in culture pretreated with LXR agonist, TO901317, exhibit improved defenses against oxidative stress generated by tert-butyl hydroperoxide, implying that LXRs play an important role in maintaining the redox homeostasis in the peripheral nervous system. Thus, LXR activation could be a promising strategy to protect from alteration of peripheral myelin resulting from a disturbance of redox homeostasis in Schwann cell.

## Introduction

Liver X Receptors (LXRα and LXRβ) belong to the nuclear receptor superfamily of ligand-activated transcription factors. They regulate target gene expression by binding to specific responsive elements and are implicated in metabolic processes such as cholesterol turnover, inflammation as well as pathologies such as cancer and neurodegenerative diseases^1^. Natural ligands of LXRs are oxysterols (*i*.*e*. 24(S)-hydroxycholesterol (24(S)-OH) or 25-hydroxycholesterol (25-OH)), produced either through auto-oxidation or enzymatic oxidation of cholesterol. Synthetic ligands of LXR, like TO901317, have also been discovered and are known to be potent activators of the LXR pathway.

Previous studies have shown that mice where both LXR isoforms (LXRα and LXRβ) are deleted (LXR double KO or LXRdKO) exhibit altered lipid homeostasis in the brain resulting in neuronal loss, astrocytic proliferation, disorganized myelin sheaths and lipid accumulation in specific brain regions that participates in locomotor defects highlighted in these animals^2,3^. We also observed that LXRdKO mice have thinner myelin sheaths surrounding axons of the sciatic nerve^4,5^. Importantly, LXR inhibition enhanced myelin gene transcripts but decreased the amount of myelin proteins, suggesting post-translational modifications, detrimental for peripheral myelin integrity.

Oxidative stress has been recently shown to alter the structure of myelin proteins in several diabetic peripheral neuropathies^6^. In particular, PMP22 misfolding and aggregation provokes demyelination and nerve conduction velocity reduction. In line with this, our team recently showed that a burst of oxidative stress induced by Paraquat provokes a dramatic alteration of myelin structure in the sciatic nerves^7^. Indeed, because of their high reactivity, reactive oxygen species (ROS) modify the structure and therefore the physiological functions of proteins and lipids. Thus, maintaining normal cellular ROS levels is essential. The excessive production of ROS or the decrease of antioxidant defenses is rather a hallmark characteristic in the pathogenesis of diseases such as diabetes, atherosclerosis and neurodegeneration^8,9^. Hence, compounds that exhibit anti-oxidative effects, triggering the intracellular cascade of protective pathways, may offer a promising strategy for therapeutic applications^10^.

Nuclear factor (erythroid-derived 2)-like 2 (Nrf2), also known as NFE2L2, is commonly involved in the transcriptional regulation of genes encoding antioxidant proteins, among which Glucose-6-phosphate dehydrogenase (G6PDH), Isocitrate dehydrogenase 1 (IDH1) and 6-phosphogluconate dehydrogenase (6-PGDH)^11^. These enzymes protect cells from oxidative stress by regenerating nicotinamide adenine dinucleotide phosphate (NAPDH). NADPH, in turn, plays a pivotal role in the regeneration of reduced glutathione (GSH), one of the most important reducers of ROS. Hence, the Nrf2 signaling pathway can be considered as a key driver of cellular antioxidant defenses. Studies have clearly shown that deletion of Nrf2 impairs functional recovery, reduces clearance of myelin debris and axonal re-myelination after peripheral nerve injury^12^. Besides, activation of Nrf2 pathway is suggested as a new therapeutic strategy for neurodegenerative diseases^13^.

The aim of this study is to understand the role of both LXR isoforms in the modulation of the redox status of myelinating Schwann cells. LXR genetic ablation provokes locomotor defects in adult mice accompanied by an elevation of oxidative stress in the sciatic nerve. The accumulation of oxidative insults (lipid peroxidation and protein carbonylation) led to myelin sheaths alterations that can be prevented by treating LXRdKO mice with the ROS scavenger N-acetylcysteine (NAC). Furthermore, we show that the LXR agonist TO901317 protects Schwann cells from ROS by enhancing the antioxidant response through Nrf2 target genes. Therefore, we highlight the potency of LXRs as new therapeutic targets to prevent oxidative stress-mediated peripheral myelin alteration.

## Results

### LXR knockout induces oxidative stress and antioxidant responses in the sciatic nerve

**Figure 1:**
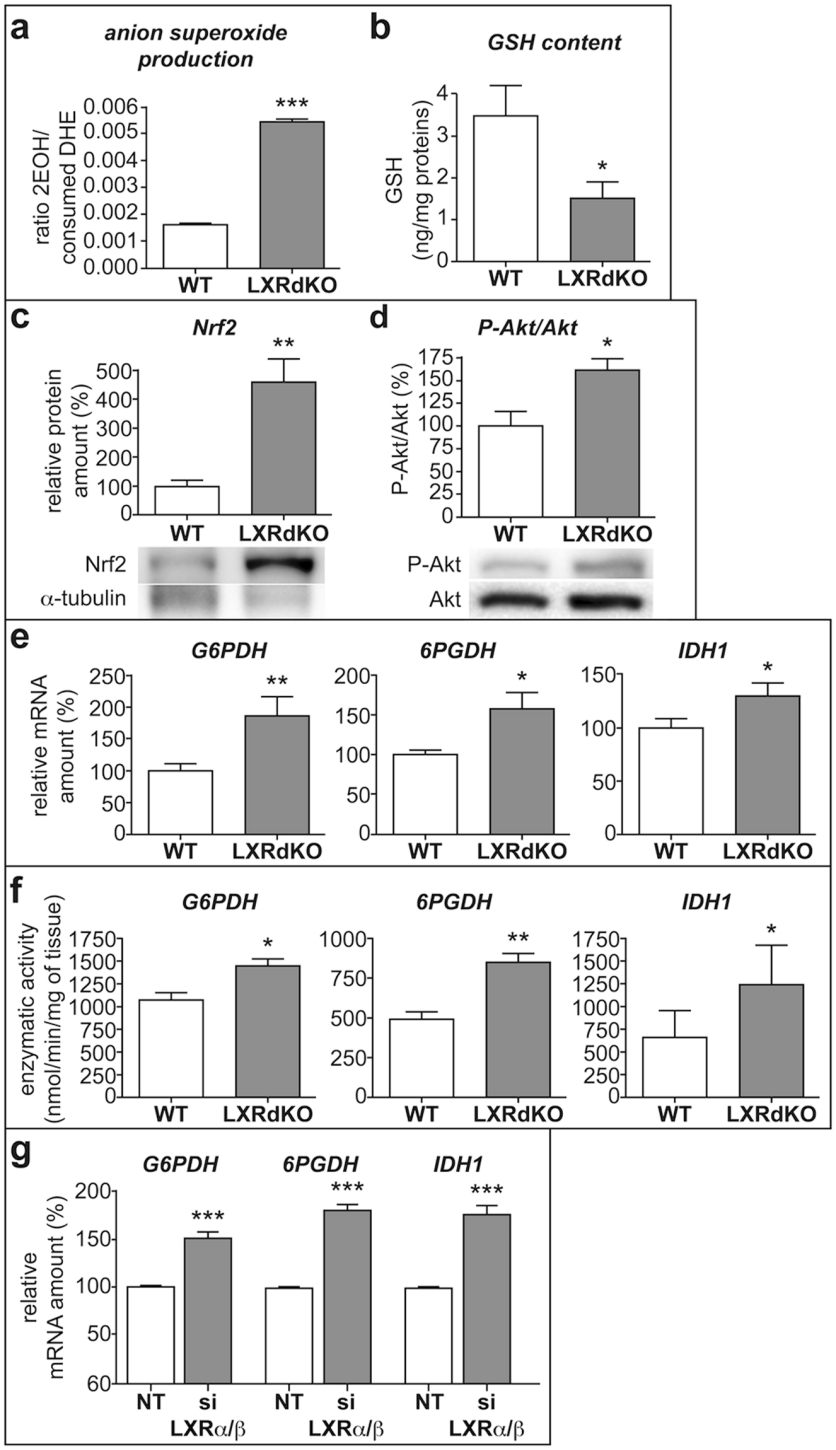
LXR ablation provokes oxidative stress in sciatic nerve. Sciatic nerves from either 8 week-old WT or LXRdKO mice were extracted. (**a**) Anion superoxide production quantified by DHE assay using HPLC. (**b**) GSH assay in WT and LXRdKO animals. (**c**) Representative western blots (cropped gels) of nerve lysate detecting Nrf2 (**d**) Akt and P-Akt proteins. All quantifications were normalized to alpha-tubulin. (**e**) mRNA quantification by RT-qPCR of antioxidant genes. Transcript levels were normalized to GAPDH expression both in control and LXRdKO animals. (**f**) Enzymatic activity assay of antioxidant genes. The activities of these enzymes were normalized to tissue weight. (**g**) mRNA levels of antioxidant response genes in MSC80 transfected with both siLXRα and siLXRß quantified by RT-qPCR. Transcript levels were normalized to GAPDH expression both in Non-Targeting controls and siLXR-transfected cells. Results represent the means ± SEM of at least three independent experiments for transfection and 5 animals per group for the other experiments. **P < 0.01 and *P < 0.05 ***P < 0.001 assessed by Student’s *t* test.

We first quantified the oxidative state in the sciatic nerves of adult LXRdKO mice. A 3-fold increase of anion superoxide production was observed (Fig. 1a) while the amount of GSH was decreased by 50% when we compared LXRdKO mice to WT (Fig. 1b). We measured the expression level of Nrf2 protein, a redox-sensitive transcription factor that controls the transcriptional activity of antioxidant enzymes. We observed a significant 2-fold increase of Nrf2 protein amount in LXRdKO nerves when compared to WT (Fig. 1c). Interestingly, we observed a parallel stimulation of the survival pathway Akt known to be an upstream effector of Nrf2 signaling^14,15^. The ratio of P-Akt/Akt was enhanced by 2.5-fold in the sciatic nerves of LXRdKO animals (Fig. 1d). Consequently, both the RNA expression level and the enzymatic activities of the major Nrf2 target genes (*i*.*e* G6PDH, 6PGDH and IDH1) were enhanced in the nerves of LXRdKO animals (Fig. 1e and f, respectively). We observed similar results *in vitro* using the mouse Schwann cell-line MSC80. The down-regulation of the expression of both LXRα and LXRβ by selective siRNA increased antioxidant gene expression by approximately 50% (Fig. 1g). Altogether, these results show that the absence of LXRs alters the redox homeostasis in Schwann cells and elicits an antioxidant response in the sciatic nerves of adult LXRdKO mice.

### Ablation of LXR does not alter myelination and redox status during development

**Figure 2:**
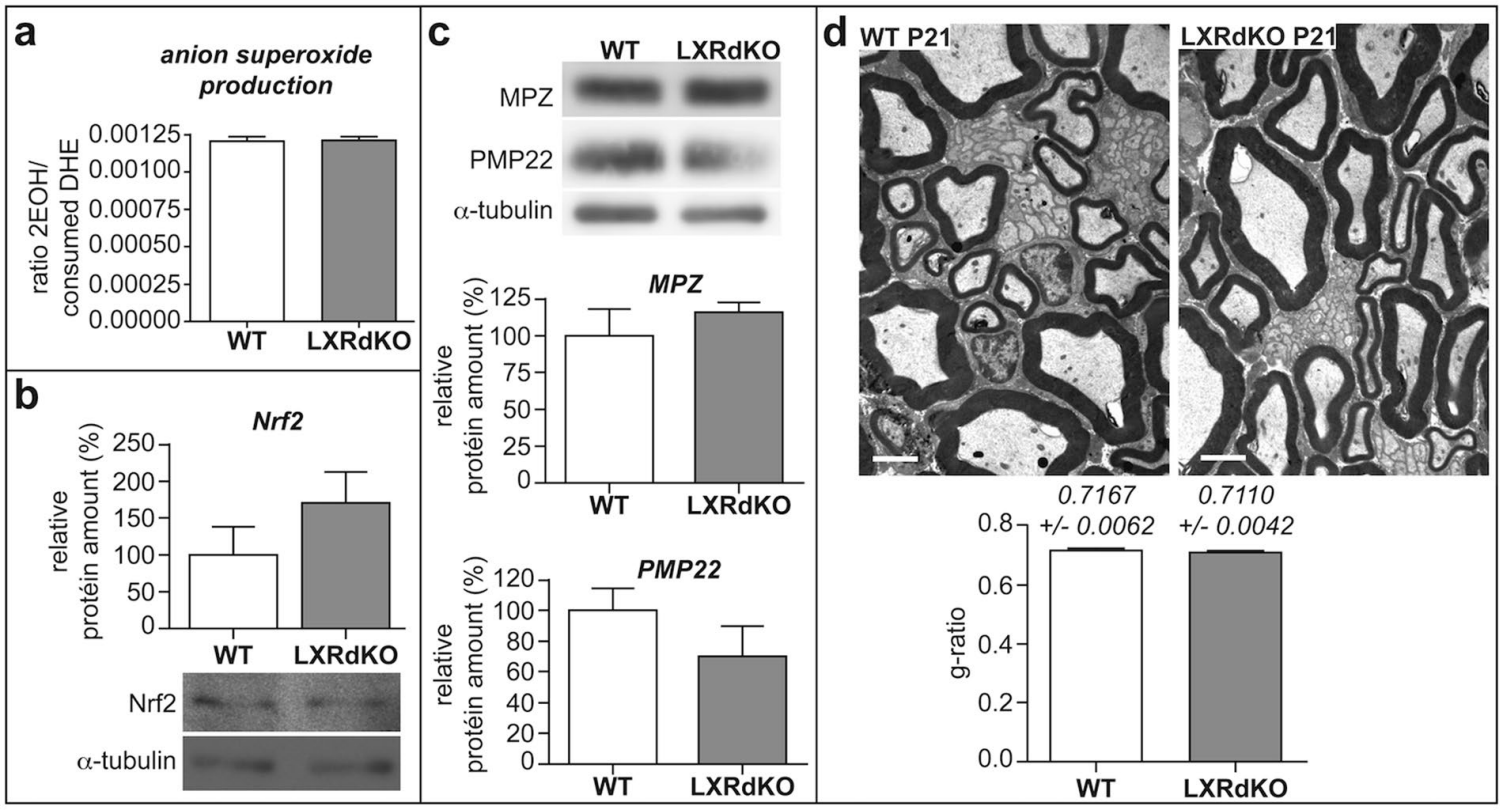
Ablation of LXR does not alter normal myelination and redox status during development. Sciatic nerves from either 21-day-old WT or LXRdKO mice were used for the experiments. (**a**) Anion superoxide production quantified by DHE assay using HPLC. Representative western blots (cropped gels) of nerve lysate detecting Nrf2 (**b**), PMP22 and MPZ (**c**) proteins. All quantifications were normalized to alpha-tubulin. (**d**) Electron microscopy images of ultrathin (50–90 nm) sciatic nerve cross-sections of WT and LXRdKO P21 mice. Myelin thickness estimated by g-ratio (inner over outer perimeter of myelinated axons). Results represent the means ± SEM of at least 5 animals per group. Statistical significance was assessed by a Student’s *t* test.

We then inquired if the knockout of LXR isoforms alters oxidative status and myelin structure from the early stages of sciatic nerve myelination. Interestingly, we observed that anion superoxide production was not affected at the end of the myelination process (post-natal day 21, P21) in the sciatic nerves of LXRdKO animals (Fig. 2a). Furthermore, Nrf2 expression was not significantly modified (Fig. 2b) and neither Myelin Protein Zero (MPZ) nor PMP22 protein amounts were altered when compared to WT mice (Fig. 2c). Finally, electron microscopy analyses of sciatic nerve ultrathin sections at P21 did not reveal any changes in myelin thickness (Fig. 2d) [calculated by g-ratio: inner over outer perimeter of myelinated axons]. These data reveal that the alteration of redox status in LXRdKO are not concomitant with myelin development but appears after the end of this process.

### N-acetylcysteine treatment markedly attenuates the myelin alteration in LXRdKO mice

Since LXRdKO exhibited myelin modifications at 8 weeks, it is likely that chronic elevation of oxidative stress leads to the accumulation of oxidative damage. Therefore, we treated LXRdKO mice with the anti-oxidant ROS scavenger N-acetylcysteine (NAC) from P21 (before the emergence of myelin abnormalities) to 8 weeks and investigated both redox status and the extent of myelin structure.

**Figure 3:**
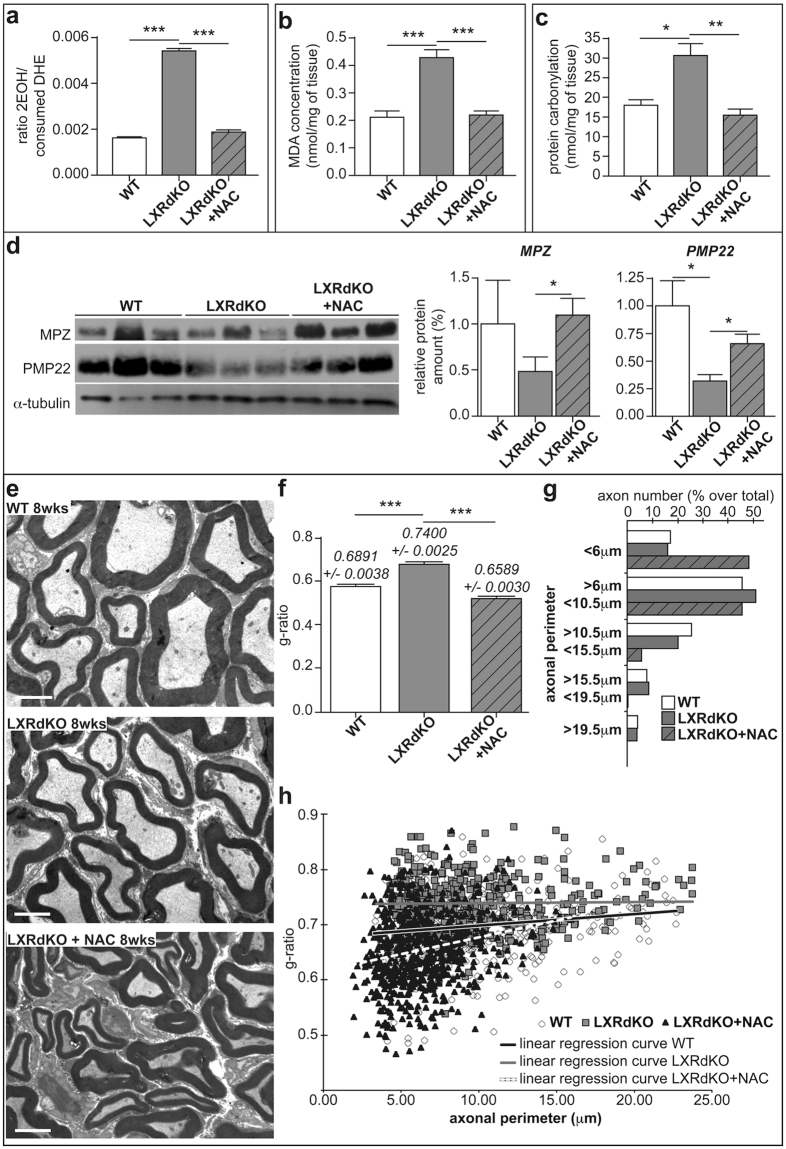
N-acetylcysteine treatment markedly attenuates myelin status alteration in LXRdKO mice. Sciatic nerves from either 8-week-old WT or LXRdKO or NAC-Treated LXRdKO mice were used for these experiments. (**a**) Anion superoxide production quantified by DHE assay using HPLC. (**b**) Lipid peroxidation assay through malonaldehyde (MDA) quantification. (**c**) Protein carbonylation assay. (**d**) Representative western blots (cropped gels) of nerve lysate detecting PMP22 and MPZ proteins. All quantifications were normalized to alpha-tubulin. (**e**) Electron microscopy images of ultrathin (50–90 nm) sciatic nerve cross-sections of WT, LXRdKO or NAC-Treated LXRdKO mice. (**f**) Myelin thickness estimated by g-ratio. Results represent the means ± SEM of at least 5 animals per group. Statistical significance was assessed by Tukey’s post *hoc test* after ANOVA. (**g**) Myelinated axon size distribution in the sciatic nerves of WT, LXRdKO and LXRdKO + NAC. (**h**) Plotted g-ratio analysis as a function of the axonal perimeter. Linear regression curve is shown in black for WT, grey for LXRdKO and white dashed line for LXRdKO + NAC animals. Results represent the means ± SEM of at least 5 animals per group. Statistical significance was assessed by a Student’s *t* test.

NAC treatment efficiently abolished oxidative stress as revealed by quantifying superoxide production by HPLC (Fig. 3a). Oxidative stress can damage nerve structure through lipid peroxidation and protein carbonylation that we assayed in the sciatic nerves by quantifying the formation of malonaldehyde (MDA) (Fig. 3b) and carbonyls (Fig. 3c), respectively. We observed that the level of MDA in the sciatic nerves of LXRdKO mice was increased by almost 2-folds when compared to WT mice. Notably, lipid peroxidation was totally reversed after NAC administration (Fig. 3b). Similarly, we found a significant increase in the overall level of protein carbonyls by almost 70% in sciatic nerve of LXRdKO mice that was reversed in the nerves of NAC-treated LXRdKO animals (17 nmol/mg of protein for WT and NAC-treated LXR dKO *vs* 31 nmol/mg protein for LXRdKO) (Fig. 3c).

Since accumulation of oxidative insults can lead to protein degradation, we assessed peripheral myelin levels in the sciatic nerves of WT, LXRdKO and NAC-treated LXRdKO mice (Fig. 3d). LXR extinction led to a decrease of both MPZ and PMP22 protein levels (−50% for MPZ, and −75% for PMP22). Interestingly, NAC treatment brought back myelin protein levels to normal. We pushed forward the analysis of the consequences of ROS generation on myelin integrity and evaluated sciatic nerve myelin ultrastructure by electron microscopy (Fig. 3e). Sciatic nerves of LXRdKO mice displayed thinner myelin sheaths as indicated by a higher g-ratio than WT mice (Fig. 3f). Interestingly, treatment of LXRdKO mice with NAC led to a significant decrease of the sciatic nerve g-ratio, bringing it even lower than in WT. LXR ablation provoked a modest shift in the size of myelinated axons (Fig. 3g), with fewer axons measuring between 10.5 and 15.5 μm of perimeter (20% over total axon number in LXRdKO animals *vs* 25% in WT animals) to the benefit of smaller ones (50% of the total axonal population sized between 6 and 10.5 μm *vs* 45% in WT). Notably, NAC administration deeply modifies axon caliber distribution in the sciatic nerves of treated animals. Indeed, high-caliber myelinated axons appear almost absent in the sciatic nerves of NAC-treated LXRdKO animals to the benefit of small-caliber myelinated axons of less than 6μm of perimeter. Despite an altered axonal caliber distribution in WT, LXRdKO and NAC treated LXRdKO animals, the total number of myelinated axons remained almost the same (16.67 + /− 5.79 for WT, 15.22 + /− 5.47 for LXRdKO and 15.55 +/− 6.42 per 500 μm^2^ for NAC Treated LXRdKO animals). Finally, the analysis of plotted g-ratio as a function of the axonal perimeter shows that the alteration of myelin thickness is more severe in small caliber axons (Fig. 3h). In addition, we also reveal that this category of smaller axons appears hypermyelinated in the sciatic nerves of NAC-treated LXRdKO animals.

### N-acetylcysteine treatment reverts the deleterious effect of LXR ablation on nerve conduction and locomotion

**Figure 4:**
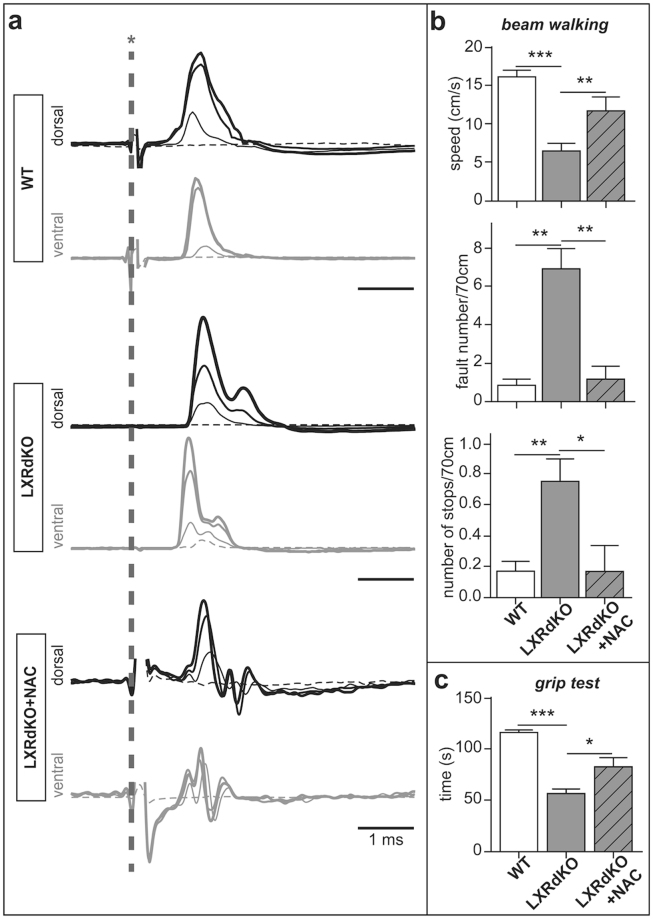
N-acetylcysteine treatment counteracts functional defects in LXRdKO mice. (**a**) Nerve conduction of WT, LXRdKO or LXRdKO + NAC animals. Representative responses recorded from L4 dorsal (top traces) and ventral (bottom traces) roots following electrical stimulation of the sciatic nerve at different stimulation intensities in WT mice, LXRdKO mice, and LXRdKO mice treated with NAC. The vertical dashed line with an asterisk indicated the time of the stimulation. Each trace is the average response (average of 10–15 sweeps) to stimulation at increasing intensities (dashed line trace, threshold intensity, thinner trace, stimulation at 1.2 × threshold, slightly thicker trace, stimulation at 1.75 × threshold, and the thickest trace is the maximum response at 2–3 × threshold). The stimulation artifacts were truncated. (**b**) Beam walking test results measuring speed, number of faults and the number of stops while moving along the beam. (**c**) Grip test results measuring the total time the mice remain suspended from a horizontal bar. Results were obtained across three trials sessions. Results are the mean +/− SEM of 5–7 animals per group; *P < 0.05; **P < 0.01; ***P < 0.001 by Tukey’s post *hoc test* after ANOVA.

We assessed the functional consequences of myelin changes in the sciatic nerves of WT and LXRdKO animals treated or not with NAC by assessing nerve conduction (Fig. 4a). On WT mice, stimulation of the sciatic nerve produced a single peak with a short latency on both dorsal and ventral roots. In LXRdKO animals, although sciatic stimulations initially generated a peak at roughly the same latency as in WT mice, they also produced a second peak at longer latency, indicating that a significant proportion of large myelinated fibers in both the dorsal and ventral roots had a reduced conduction velocity compared to controls. Treatment with NAC had complex effects on LXRdKO mice, with three peaks appearing on the response to sciatic stimulation. Although the first peak retained roughly the same latency as in the controls and LXRdKO mice, the second peak tended to have a slightly shorted latency than the second peak observed in LXRdKO mice. Furthermore, a third peak was visible, with a longer latency than both preceding peaks. We think that this third peak corresponds to fibers that, in LXRdKO mice, were conducting too slowly to be synchronized and were therefore unable to produce a measureable peak in the response. These fibers appeared to become faster and more synchronized due to the protective effects of NAC.

Peripheral myelin alterations also led to severe locomotor deficits and muscular weakness that were evaluated using the beam walking test and the grip test, respectively (Fig. 4b and c). We analyzed the ability of WT and LXRdKO animals treated or not with NAC to remain upright and to walk on an elevated and narrow beam. We observed that LXRdKO mice walked the distance twice slower than the control animals (15 cm/sec for WT mice *vs* 7 cm/sec for LXRdKO mice). Interestingly, NAC-treated animals crossed the beam twice faster than the non-treated LXRdKO mice. Moreover, the number of foot slips made by LXRdKO animals was significantly increased by 6-fold when compared to WT mice, but was brought back to normal following NAC treatment. Furthermore, we noted a 3-fold augmentation in the number of stops made by LXRdKO animals that was decreased to WT level after NAC administration (0.2 stop/70 cm for WT and NAC-treated LXRdKO *vs* 0.7 stop/70 cm for LXRdKO). Finally, we evaluated the muscular strength and fatigue using the grip test and showed that the NAC-treated LXRdKO mice were able to hold on to the rod using their forelimbs much longer than the LXRdKO mice (Fig. 4c). Indeed, while WT mice succeeded to hold on for over 120 seconds, LXRdKO mice remained suspended for less than 50 seconds while the NAC-treated LXRdKO animals were able to hold on for about 80 seconds.

These results point-out that the absence of LXRs in mice leads to increased ROS production in the sciatic nerve that alters cellular constituents and compromises myelin integrity. This oxidative stress participates in electrophysiological and locomotion defects that could be reversed by treating the mice with N-Acetylcysteine.

### LXR activation by TO901317 regulates anti-oxidant enzyme expression in Schwann cells

**Figure 5:**
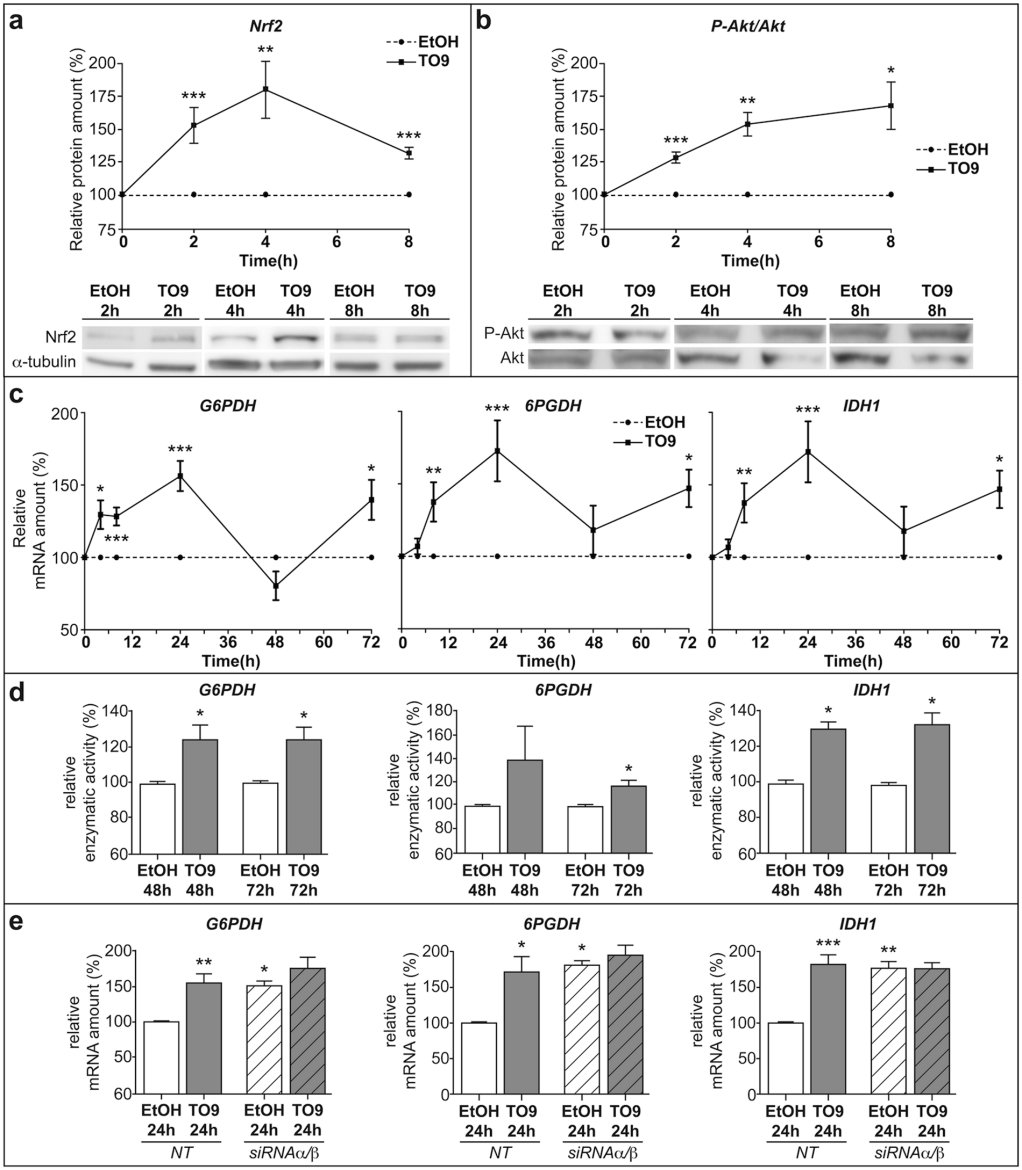
LXR activation by TO901317 regulates anti-oxidant enzyme expression in Schwann cells. MSC80 cells were treated either with TO9 (10 µM) or vehicle (Ethanol) for 2 h, 4 h, and 8 h. Total proteins were extracted at the specified time points followed by Western Blotting. (**a**) Expression profile of Nrf2 protein quantified through western blots (cropped gels). All blots were normalized to alpha-tubulin expression. (**b**) Phosphorylation profile of Akt protein (Ratio P-Akt/Akt) quantified through western blots (cropped gels). All blots were normalized to alpha-tubulin expression. (**c**) mRNA expression profile of antioxidant response genes across 72 h. Transcript levels were normalized to GAPDH expression. Results represent the means ± SEM of at least three independent experiments. *P < 0.05, **P < 0.01 and ***P < 0.001 assessed by Tukey’s post *hoc test* after ANOVA. (**d**) Enzymatic activity of antioxidant response genes at 48 h and 72 h. Enzymatic activity is normalized to tissue weight. (**e**) MSC80 cells were transfected with either non-target siRNA (NT) or siRNA directed against LXRα and LXRß. 48 hours after transfection, cells were treated either with T09 (10 µM) or vehicle (Ethanol) for 24 h. mRNA levels of G6PDH, 6PGDH and IDH1 were assessed through RT-qPCR and normalized to GAPDH expression. Results represent the means ± SEM of at least three independent experiments. *P < 0.05, **P < 0.01, ***P < 0.001 assessed by Student’s *t* test.

Our findings indicate that LXRs signaling could be a major actor in the modulation of redox status in the nerve and suggest its relevance for the treatment of oxidative stress related disorders. Consequently, we questioned whether LXR activation by TO901317 (TO9) could modulate Nrf2 signaling in a mouse Schwann cell-line (MSC80). Western-blot experiments showed that Nrf2 protein levels increased after TO9 treatment (between 2 h to 8 h) (Fig. 5a). Akt survival pathway, upstream effector of Nrf2, was also activated after incubation with TO9. Indeed, Akt phosphorylation was increased by 25% after 2 h, and reached its sustained maximum activation level after 4 hours until at least 8 hours of TO9 treatment (Fig. 5b).

Nrf2 activation by TO9 is followed by the stimulation of anti-oxidant genes: G6PDH, 6PGDH and IDH1 (Fig. 5c). TO9 treatment caused a time-dependent increase of G6PDH, 6PGDH and IDH1 transcripts after 4 and 8 h of treatment, with a peak activity seen at 24 h with 60% increase. An induction in the enzymatic activities of G6PDH, 6PGDH and IDH1 was observed (Fig. 5d). Interestingly, the knockdown of LXRs by siRNA in MSC80 abrogated the activation of antioxidant genes by TO9 (Fig. 5e), confirming that TO9-mediated antioxidant response in Schwann cells requires the presence of LXRs. Altogether these results show that TO9 can trigger cellular antioxidant responses and could be potent enough to alleviate ROS-mediated deleterious outcomes in Schwann cells.

### LXR activation reduces ROS generation and cytotoxicity *in vitro*

**Figure 6:**
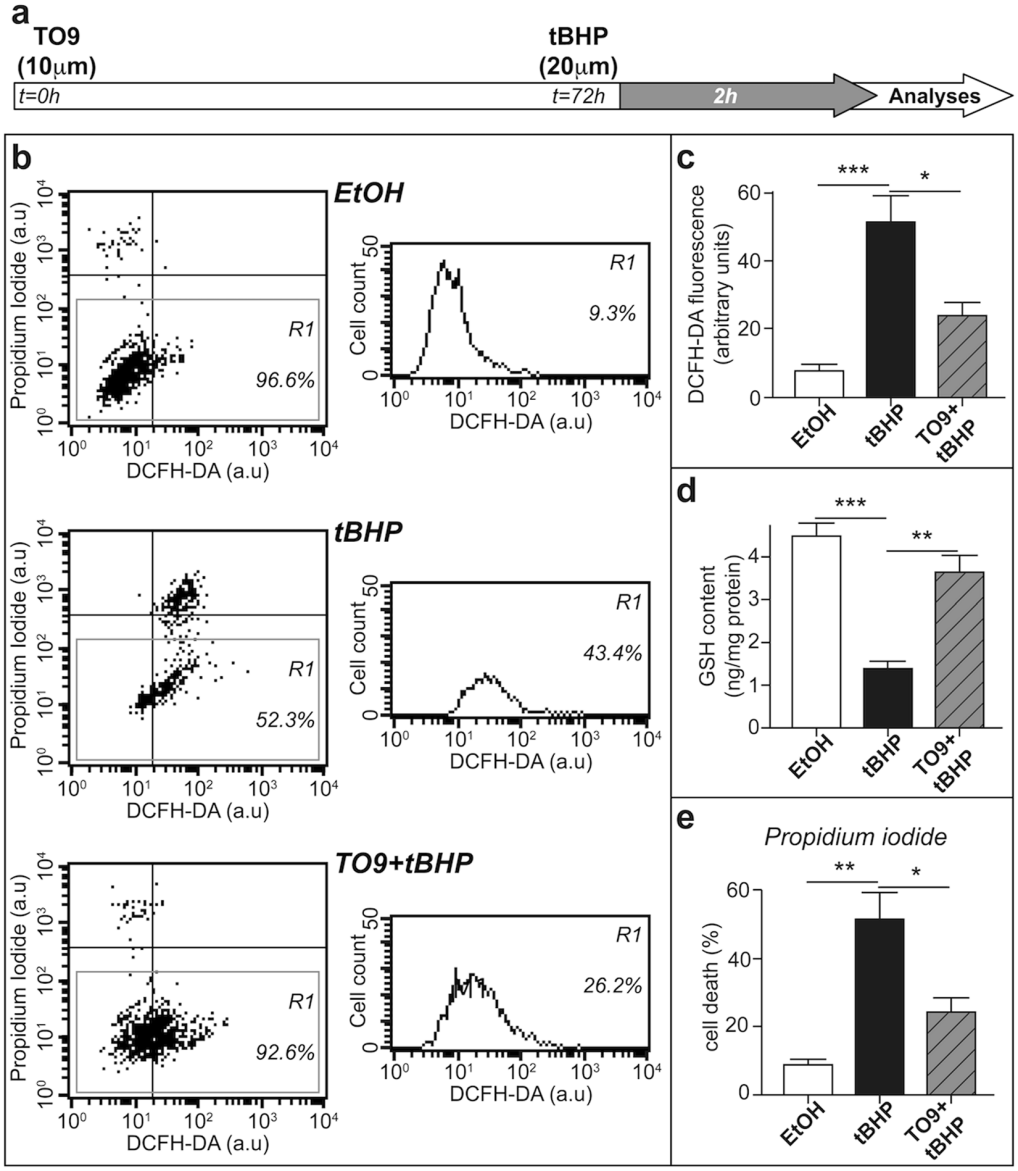
LXR activation reduces ROS generation and cytotoxicity *in vitro*. MSC80 cells were pre-incubated with either TO9 or vehicle control for 72 h and then subjected to tert-butyl hydroperoxide (tBHP) treatment during 2 h. (**a**) Schematic time scale representation of the experiment performed. (**b**) Representative FACS analysis results for each condition (Vehicle control Ethanol: EtOH, Tertbutyl hydroperoxide: tBHP and TO9 + tBHP treatment) X axis represents DCFH-DA fluorescence and Y-Axis represents PI fluorescence. The scatter plot represents the entire population of cells with their respective DCFH-DA and PI fluorescence. The R1 gate considers only live cells to quantify DCFH-DA fluorescence and excludes dead cells. (**c**) Oxidative stress assessed by DCFH-DA fluorescence across 3 individual FACS experiments. (**d**) GSH assay results after tBHP treatment. Cell death assessed by PI fluorescence across 3 individual FACS experiments. Results are presented as the mean+/− SD values of three independent experiments. *P < 0.05, **P < 0.01, ***P < 0.001 by Tukey’s post hoc test after one-way ANOVA when compared with control.

We finally investigated whether LXR activation by TO9 could protect Schwann cell-line from the deleterious effects of increased ROS production. We pre-treated MSC80 with TO9 for 72 h before provoking ROS production by *tert*-butyl hydroperoxide (tBHP) (Fig. 6a). We quantified ROS production and cell death by flow cytometry (using DCFH-DA and propidium iodide staining, Fig. 6b) and observed that TO9 pre-treatment was able to dampen ROS production (Fig. 6c). The cells appear to be well prepared to fight against the oxidative stress. Indeed, the amount of GSH was decreased by almost 60% after tBHP treatment, and that TO9 pre-treatment brought back GSH concentration to control levels (Fig. 6d). Moreover, TO9 treatment is able to prevent cell death elicited by tBHP treatment (Fig. 6e) as observed through decreased PI florescence.

## Discussion

LXR activation is classically known to have beneficial outcomes for the treatment of several chronic diseases through anti-inflammatory effects and regulation of cholesterol homeostasis^16–20^. For example, LXR activation affects Parkinson disease progression by modulating the cytotoxic functions of microglia^21^ and attenuates diabetic neuropathy by stimulating neuroprotective steroid synthesis^16^. LXRs have also been exploited as potential targets for oxidative stress disorders that provoke myocardial dysfunctions in diabetic db/db model mice^22,23^.

In this study, we established that LXRs have an anti-oxidant potential in Schwann cells and in the peripheral nerve. We detected an increased anion superoxide production in the sciatic nerves of LXRdKO mice. We also observed a concomitant augmentation in lipid peroxidation and protein carbonylation along with a drop in reduced glutathione, hallmarks of many chronic neurodegenerative diseases^24^. Previously, our team showed that the knock-out of LXRs reduced the thickness of myelin sheaths in the sciatic nerves of adult animals^4,5^. This phenotype was accompanied by a down regulation of myelin protein levels despite a stimulation of their respective transcripts. Here, we provide evidence that the myelin disorder observed could be linked to altered redox status in the sciatic nerves of LXRdKO animals, leading to accumulation of oxidative insults responsible of peripheral myelin progressive alteration. Indeed, in young LXRdKO animals (P21) neither the alteration of myelin structure nor the activation of cellular responses to oxidative stress was detectable. Thus, the compromised myelin integrity observed in adult LXRdKO mice is not initiated at birth but develops after the end of the myelination process (P21). The oxidative environment produced by the loss of LXR function coupled with an overexpression of myelin genes can presumably induce accumulation of modified cellular constituents as the animal grow and progressively compromises myelin integrity. This plausible cause-effect explanation is indeed verified when we administered the antioxidant molecule, NAC, to LXRdKO mice from P21 until P56 (8 week-old). A 5-week long treatment with this ROS scavenger allowed the mice to recover from locomotor impairment elicited by LXR genetic ablation, together with a normalization of both sciatic lipid peroxidation and protein carbonylation levels.

Besides, NAC treatment led to profound changes in axonal topology within the sciatic nerves of LXRdKO animals. A dramatic increase of the number of small and hypermyelinated axons were observed at the expense of large caliber axons that were missing. In fact, we previously demonstrated that LXRs signaling is a negative driver of myelination in the PNS^4,5^. Thus, myelin genes are stimulated in the absence of LXRs. Attenuation of the oxidative stress due to NAC administration prevented the accumulation of oxidative insults that could participate in myelin degradation, thereby inducing hypermyelination of certain type of axons. The shift in sciatic axonal topology was unraveled when we analyzed the nerve conduction velocity. LXRdKO animals exhibited faults in transmitting rapid nervous influx accounting for their locomotor defects. NAC-treated animals exhibited a more complex scheme, with the appearance of shifted faster peaks that could compensate for the loss of large, fast axons, through the recruitment of small, hypermyelinated axons. Those could allow a faster transmission of the influx and improve fine locomotion behavior we highlighted in NAC-treated LXRdKO animals. Altogether, we have shown here that the locomotor defects observed in LXRdKO mice could be reversed upon an antioxidant treatment with NAC from P21, before the emergence of oxidative stress and alteration of myelin status. These observations underline the deleterious consequences of the accumulation of oxidative damage^25,26^. Moreover, the functional, cellular and molecular recovery initiated by NAC suggest a direct link between increased ROS production and myelin damage accumulation in the peripheral nerves in the absence of LXRs. However, these alterations in myelin status could also be attributed to axonal diameter changes. Indeed, several signaling pathways govern the tight crosstalk between Schwann cells and axons. For instance, it is well known that axonal Neuregulin-1 interacts with ErB receptor on myelinating Schwann cells to command myelin thickness^27^. In fact, the NAC treatment leading to a shift in sciatic axonal topology could in turn affect myelination.

Dynamic changes are seen in the Nrf2 pathway in patients as well as in animal models of aging and disease^28^. Akt/Nrf2 pathway has already been implicated in neuroprotection against oxidative stress damage in the CNS. Indeed, Lee and colleagues showed that an inhibition of the molecular hub PI3K/Akt pathway provokes the downregulation of Nrf2 signaling and subsequent oxidative stress in mouse hippocampal HT22 cells^29^. In LXRdKO nerves, enzymes implicated in glutathione metabolism are stimulated, but the extent of endogenous activation of Nrf2 is not sufficient to combat the overload of oxidative stress. Interestingly, both LXR inhibition in mice and LXR activation by TO9 in Schwann cells stimulated Nrf2 signaling through Akt survival pathway. These results are in accordance with recent studies showing that exogenous activation of Nrf2 pathway has neuroprotective effects in fighting against sciatic nerve oxidative stress damage in streptozotocin-induced diabetic rats^30,31^. Moreover, deletion of Nrf2 also impairs functional recovery, reduces clearance of myelin debris and decreases axonal remyelination after peripheral nerve injury^12^.

Furthermore, when MSC80 cells were treated with LXR agonist, TO9, we observed a stimulation of the enzymatic antioxidant response sufficient enough to counteract the deleterious effects of *tert*-butylhydroperoxide, a potent pro-oxidant molecule. Hence, following LXR activation in Schwann cells, we were able to stimulate antioxidant defenses to dampen ROS production and subsequent alteration of cellular constituents causing cell death.

We previously revealed that LXR agonists are encouraging candidates for the treatment of central myelin disorders as they were able to stimulate myelin gene expression and cellular maturation to increase remyelinating efficacy^3^. Here we show that LXR activation could also be a promising strategy to reduce oxidative stress-induced damages by activating an anti-oxidant response in the peripheral nervous system. Therefore, this study helps to better understand the importance of LXRs in the modulation of cellular redox status in the peripheral nerve and paves way for developing promising avenues for the treatment of oxidative stress disorders based on LXR modulation.

## Methods

All methods were performed in accordance with the relevant guidelines and regulations of INSERM and Université Paris Descartes.

### Animals and *in vivo* experiments

LXRdKO mice and their wild-type (WT) controls were maintained on a mixed strain background (C57BL/6:129 Sv) and housed in a temperature-controlled room with a 12 h light/dark cycle. All experiments were performed on age-matched male mice (8 weeks old). Animals were fed ad libitum with water. N-acetylcysteine (NAC) treatment was administrated in a 0.1% drinking water solution during five weeks to independent groups of LXRdKO mice from P21 to P56 (8 week-old mice). Sciatic nerves were collected and frozen either in liquid nitrogen or isopentane. All aspects of animal care were approved by the National Ethic Committee (No. 2016092216181520).

### Cell culture and treatment

The mouse SC line (MSC80) was maintained in Dulbecco’s minimal essential medium (DMEM) supplemented with 10% decomplemented fetal calf serum (Hyclone-Perbio), 1% penicillin, 1% streptomycin (Gibco) and 1% Glutamine (Gibco). All cultures were grown at 37 °C in a humidified atmosphere of 5% CO2. MSC80 cells were treated with TO901317 (Tocris) at the concentration of 10 µM in DMEM.

### Transient transfections

Cells were transiently transfected with siLXRs (Dharmacon) using Effecten reagent (Effecten Transfection Reagent, Qiagen). One day prior to the transfection, MSC80 cells (1.5 × 10^5^ cells/well) were cultured on 6-well plates and incubated in the DMEM culture medium containing 10% heat inactivated fetal calf serum. Sixteen hours post transfection, the medium was replaced by DMEM or DMEM containing TO901317 (10 μM) or vehicle (Ethanol).

### Behavioral tests

#### Rod test

Mice were trained to walk along a horizontal rod (70 cm long, 3 cm diameter) placed at an elevation of 30 cm on a bench. The mice were taught to move from a platform on one end to the goal box on the other. Once the mice had succeeded in reaching the goal box several times with the help of investigator, the test was recorded in three trials. Mice that fell were returned to the position they fell from with a maximum time of 60 s allowed on the beam. The speed, the number of times the mice slipped on the rod, the number of falls and stops were measured. A high definition digital camera was used to film the experiment.

#### Grip test

The time spent holding onto a thin metal rod suspended in midair was calculated. Each mouse was subjected to 3 successive attempts separated by a 10-minute rest period. All tests were made blind, the group assignment being unknown to the observers.

### Sciatic nerve conduction velocity measurement

8 week-old mice were anesthetized with isofluorane. Once surgical plane was reached, the mouse was transferred to a heating pad and anesthesia was continued through a gas mask. The right sciatic nerve was dissected free, cut distally, and placed on a bipolar stimulation electrode. A laminectomy was then performed on vertebral segments L1-L5. The L4 dorsal ganglion was identified and the L4 dorsal and ventral roots were dissected and cut close to the spinal cord. Each root was placed on a recording electrode. The sciatic nerve was stimulated at 1 Hz at increasing stimulation intensities while afferent and antidromic efferent volleys were recorded on the dorsal and ventral root, respectively. At the end of the experiment, mice were killed with an overdose of pentobarbital.

### Lipid peroxidation

Lipid peroxidation assay kit (Abcam ab118970) was used to detect malonaldehyde (MDA) present in samples as per the manufacturer’s instruction with slight modifications. Detailed description can be found in Supplemental Methods.

### Protein carbonylation

Sciatic nerves were homogenized in 5–10 ml of 100 mM Tris, pH 7.4 and then, tissues were centrifuged at 10000 g for 15 min at 4 °C. the supernatant was removed and stored on ice. Protein concentration of each sample was determined by DC assay (BIORAD) and adjusted between 5–10 mg/ml. 200 μl of each sample was mixed with 200 μl of 10 mM DNPH in 2 N HCl or with 200 μl of 2 N HCl alone for the blank and incubated at room temperature for 1 h in the dark. Samples were frequently vortexed every 15 min. Proteins were precipitated with 20% trichloroacetic acid (w/v), vortexed and centrifuged (13,000 g for 3 min). The pellet was washed three times with 1 ml ethanol-ethyl acetate (1:1 v/v) before re-dissolving in 1 ml of 6 M guanidine HCl in 20 mM potassium phosphate adjusted to pH 2.3 with trifluoroacetic acid. The absorbance was measured in the supernatant at 360 nm and carbonyl content was calculated, using the molar absorption coefficient of 22,000 M^−1^cm^−1^ relative to protein concentration.

### GSH measurement

Intracellular reduced glutathione (GSH) levels from sciatic nerves and MSC80 cells were measured by the Glutathione assay kit (Sigma Aldrich CS1020) as per the manufacturer’s instructions.

### Western blot

Frozen sciatic nerves from adult mice were homogenized using a Bead Mill Homogenizer (RETSCH MM300) in RIPA buffer [25 mM Tris•HCl pH 7.6, 150 mM NaCl, 1% NP-40, 1% sodium deoxycholate, 0.1% SDS] supplemented with Protease Inhibitor Cocktail. (Roche) The lysates were centrifuged at 12000 g for 15 min at 4 °C and protein content in the supernatant was assessed using the RC DC protein assay kit (Bio-Rad) with BSA (2 mg/ml) as standard. Equal quantities of Sciatic Nerve or MSC80 protein lysates were separated on 12% SDS-PAGE and blotted onto polyvinylidene difluoride (PVDF) membranes. Nonspecific binding sites in the transblots were blocked with 5% Bovine Serum Albumin (Sigma) in Tris Buffer Saline 1 × with 0.1% Tween 20 (Invitrogen) for at least an hour. Membranes were then incubated at 4 °C overnight with the following primary antibodies diluted in a mixture of BSA 5%/TBS- Tween 0.1%: Rabbit polyclonal PMP22 antibody (Abcam, 1:1000), P0 antibody (Abcam, 1:750), Nrf2 antibody (Abcam, 1:100), Phospho-Akt Serine 473 antibody (Abcam, 1:1000), Akt antibody (Abcam, 1:1000), α-tubulin antibody (Abcam, 1:10000). The membranes were then washed and incubated at room temperature for 1 h with the appropriate secondary antibody (HRP conjugated Goat Anti-Mouse or Anti-Rabbit IgG at 1:20000, Millipore) diluted in 5% BSA/TBS–0.1% Tween followed by ECL Plus Western Blot Detection (GE Healthcare). Protein bands were detected using ImageQuant LAS 4000 imager (GE healthcare) and quantified using NIH Image J Software.

### Transmission electron microscopy

Eight-week-old and P21 mice were deeply anesthetized by intraperitoneal injection of 40 mg/kg ketamine and 30 mg/kg xylazine and then intracardially perfused with 4% paraformaldehyde, 2.5% glutaraldehyde, and 0.1 M phosphate buffer, pH 7.4. Tissues were dissected and immersed in the same fixative solution at 4 °C overnight, washed in phosphate buffer, postfixed in 2% osmium tetroxide, dehydrated in graded ethanol series and embedded in epoxy resin. For electron microscopy, ultrathin sections (50–90 nm) were cut on an ultramicrotome (8800 Ultrotome III; LKB Bromma) and collected on 300-mesh nickel grids. Staining was performed on drops of 4% aqueous uranyl acetate, followed by Reynolds’s lead. Ultrastructural analyses were performed in a JEOL jem-1011 electron microscope and digitalized with DigitalMicrograph software.. Electron microscopy images were used for calculating the g-ratio and axon perimeter using NIH ImageJ software. At least 100 randomly selected axons were analyzed per animal. At least three animals were used per genotype. Healthy axons were defined based on the presence of intact membranes and the normal complement of organelles.

### RT-qPCR experiments

Total RNA from sciatic nerves or cultured MSC80 was obtained using TRIzol Plus RNA purification Kit (Thermo Fischer Scientific). One microgram was reverse transcribed with random primers (Promega) and reverse transcriptase MMLV-RT (Invitrogen). PCR experiments were performed using TaqDNA polymerase (Promega) and specific primer sequences (Eurofins). Quantitative real-time PCR was performed with standard protocols using SYBRGreen ROX Mix (ABgene) as a fluorescent detection dye in ABI PRISM 7000. For a final reaction volume of 7 μl, 300 nM primers and 20 ng of reverse-transcribed RNA were used in 384-well plates. Each reaction was performed in triplicate, and the mean of at least three independent experiments was calculated. The melt curve analyses were used to characterize amplicons and to check for contamination. Relative mRNA quantities were normalized to the GAPDH mRNA level and expression fold change was calculated using the ΔΔCt method. Refer to Supplementary Table 1 for primer sequences.

### Enzymatic activity assays (G6PDH, 6PGDH, IDH assay)

Frozen cells or sciatic nerves were collected and homogenized in 50 µl of resuspension buffer. 3 µl of each sample were added to 200 µl of either:

(i) G6PDH buffer (100 mM AMP-OL pH 9.4, 1 mM of Glucose-6-Phosphate, 50 µM of NADP + , 0,5 mM of EDTA and 0.02% BSA)
(ii) 6PGDH buffer (50 mM Tris acetate buffer pH 8.1, 1 mM of 6-phosphogluconate, 50 µM of NADP + , 0,1 mM of EDTA and 0.05% BSA)
(iii) IDH buffer (50 mM Tris HCl buffer pH 8.2, 2 mM of isocitrate 1 mM of NADP + , 0,2 mM of MnCl_2_ and 0.05% BSA)

In these assays, enzymatic activities are proportional to the concentration of NADPH produced from NADP + reduction^32^. The fluorescence signal was read every 2 minutes for a total time of 20 minutes in a plate reader (TECAN) at Ex/Em = 340 nm/460 nm. Enzymatic activity is expressed in nanomoles of NADPH produced per min normalized to tissue weight.

### Assessment of cytosolic ROS and cell death using flow cytometry

Flow cytometry experiments were performed on a FACS Calibur flow cytometer (Becton Dickinson, San Jose, CA, USA). Cellular oxidative stress was measured by flow cytometry using dichloro-dihydro-fluorescein diacetate (DCFH-DA) assay (Molecular Probes by Life technologies, D-399). MSC80 cells (250000 cells/well in 6-well plates) were pretreated with T0 for 72 h followed by 2 h of tert-butyl hydroperoxide (tBHP 20 µM) treatment to induce ROS production. After tBHP treatment, the cells were washed, collected and incubated with 0.5 µM DCFH-DA for 30 min at 37 °C. After DCFH-DA incubation the cells were centrifuged and resuspended again in 1 ml of PBS with 1 µl of PI solution (50 µg/ml, Life technologies) followed by flow cytometry. A total of 10.000 events were recorded with a flow rate of less than 200 cells/second for each assay. Data analysis was performed using BD Cell Quest Pro Software.

### *In situ* detection of superoxide by HPLC

HPLC experiments were performed on Jasco HPLC system, LC-2000 plus series as previously described^10,11^. For a detailed protocol refer to supplementary methods.

## Electronic supplementary material

Supplementary Information
