# Supplementary material for: Liver X Receptor exerts a protective effect against the oxidative stress in the peripheral nerve
Source: Sci Rep. 2018 Feb 6;8:2524. doi: 10.1038/s41598-018-20980-3 (PMC5802790; doi:10.1038/s41598-018-20980-3)
Supplement: Supplementary file 1 — Supplementary Information [file 41598_2018_20980_MOESM1_ESM.doc]

**Liver X Receptor exerts a protective effect against the oxidative stress in the peripheral nerve**

Mehdi Hichor^1^, Venkat Krishnan Sundaram^1^, Stéphanie A Eid^1^, Ronza Abdel-Rassoul^1^, Patrice X Petit^1^, Didier Borderie^1^, Jean Bastin^1^, Assaad A Eid^2^, Marin Manuel^3^, Julien Grenier^1^ and Charbel Massaad^1^*

^1^ Paris Descartes University, INSERM UMR-S 1124, Faculty of Basic and Biomedical Sciences, 45 rue des Saints-Pères, 75270, Paris Cedex 6, France

^2^ American University of Beirut, Department of Anatomy, Cell Biology and Physiological Sciences, PO Box 11-0236, Riad El-Solh, 1107 2020, Beirut, Lebanon, Beirut, Lebanon.

^3^ Centre de Neurophysique, Physiologie et Pathologie, Université Paris Descartes, CNRS UMR 8119, Paris, France.

**^*^ Corresponding author:**

Charbel Massaad, INSERM UMR-S 1124, Paris Descartes University*, Sorbonne Paris Cité University, Faculté des Sciences Fondamentales et Biomédicales,*

45 rue des Saints-Pères, 75006 Paris, FRANCE.

charbel.massaad@parisdescartes.fr

Phone: + 33 1 42 86 22 22

**Supplementary Information**

**Supplementary Methods**

**Lipid peroxidation assay**

The free MDA generated during lipid peroxidation refers to the oxidative degradation of lipids reacts with Thiobarbituric Acid (TBA) to generate a MDA-TBA adduct. This assay kit detects MDA levels as low as 1 nmol/well colorimetrically (OD = 532 nm). Sciatic nerves from WT and LXRdKO mice treated either with NAC or vehicle (control) were collected in 303 µl of MDA lysis solution using a Dounce homogenizer (10-50 passes) precooled on ice. Samples were then centrifuged at 13.000g for 10 min. and total protein content was quantified using RCDC Assay (BIORAD). To generate MDA-TBA adduct, 600µl of TBA reagent was added into each vial containing the supernatant previously collected. Samples were then incubated at 95°C for 60 min. and subsequently cooled in an ice bath for 10 min. Each 800µl of TBA/sample and standard mix (for the standard curve calculation) were added in a 96-wells plate (BD science). The absorbance of TBA-MDA adduct was measured at 532 nm. The MDA concentration in standard and samples was determined from their absorbance as per the manufacturer’s instructions. (Abcam ab118970)

**HPLC:**

Freshly isolated nerves are stroked with mortar and pestle suspended in 40 μl of acetonitrile. The solution is then centrifuged at 12,000 g for 10 min at 4°C. The homogenate was dried under vacuum and analyzed by Quantification of DHE, EOH, and ethidium concentrations was performed by comparison of integrated peak areas between the obtained and standard curves of each product under identical chromatographic conditions EOH and ethidium were monitored by fluorescence detection with excitation at 510 nm and emission at 595 nm, whereas DHE was monitored by ultraviolet absorption at 370 nm.

**Supplementary Table 1**

List of Primers used in qPCR experiments

| G6PDH F | CAGAGCAGGTGACCCTAAGC |
| --- | --- |
| G6PDH R | GCATAGCCCACAATGAAGGT |
| 6PGDH F | GGGCACTTTGTGAAGATGG |
| 6PGDH R | AACAGCTCTTTGCCGTCAGT |
| IDH1 F | AGGTTCTGTGGTGGAGATGC |
| IDH1 R | GACGCCCACGTTGTATTTCT |
| GAPDH F | GGTCCTGAGTTCAAATCCC |
| GAPDH R | GCGCAAGCAGGTCTGAATCGTG |
| LXRα F | CAATGCCTGATGTTTCTC |
| LXRα R | TGACTCCAACCCTATCCCTA |
| LXRβ F | AAGCAGGTGCCAGGGTTCT |
| LXRβ R | TGCATTCTGTCTCGTGGTTGT |
| Nrf2 F | CTTCCATTTACGGAGACCCACC |
| Nrf2 R | GGATTCACGCATAGGAGCACTG |

**Full length Blots:**

**Figure 1: LXR ablation provokes oxidative stress in sciatic nerve**

The images below are the full-length blots used for the quantification of Nrf2, Akt and P-Akt in Figure 1.


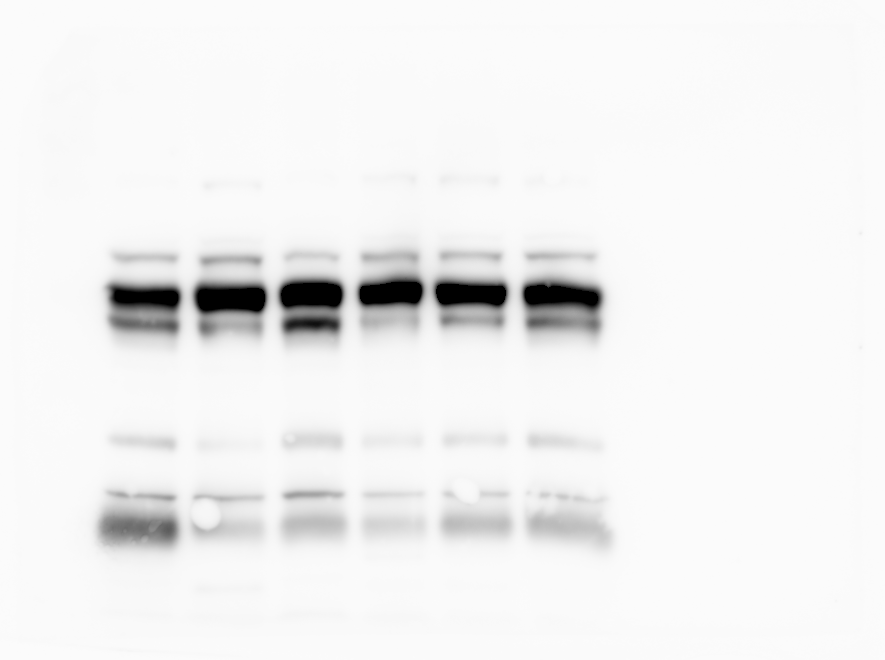


NRF2

P-Akt

A-tub

WT

WT

dKO

dKO

dKO

dKO


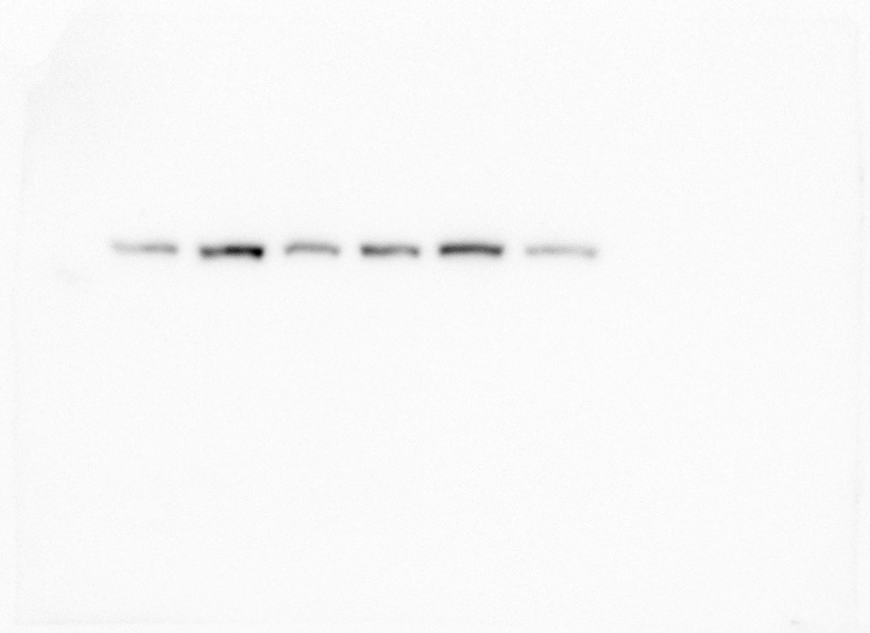


Akt total

WT

WT

dKO

dKO

dKO

dKO


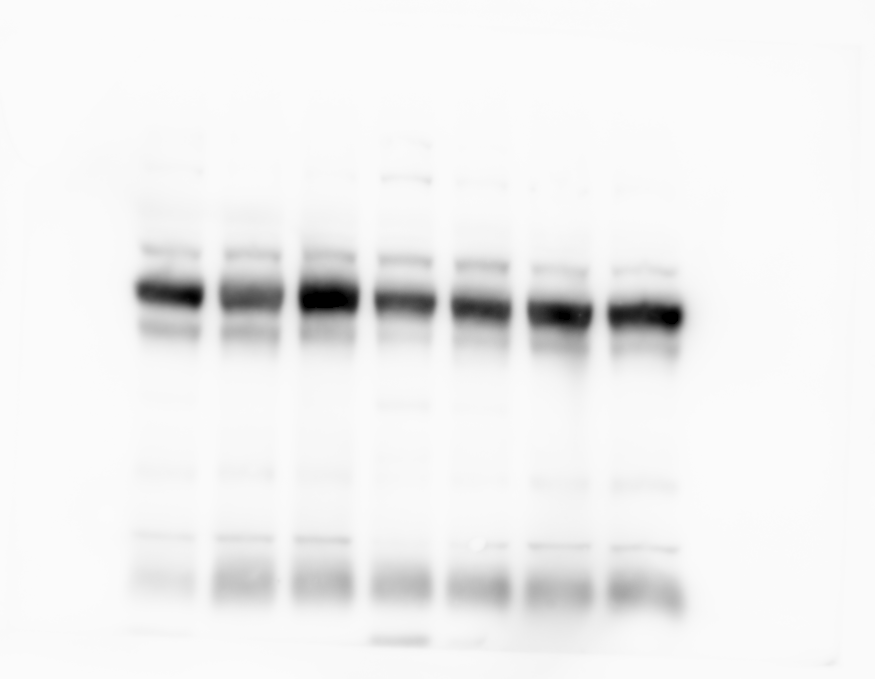


NRF2

P-Akt

A-tub

WT

WT

dKO

dKO

dKO

dKO

WT


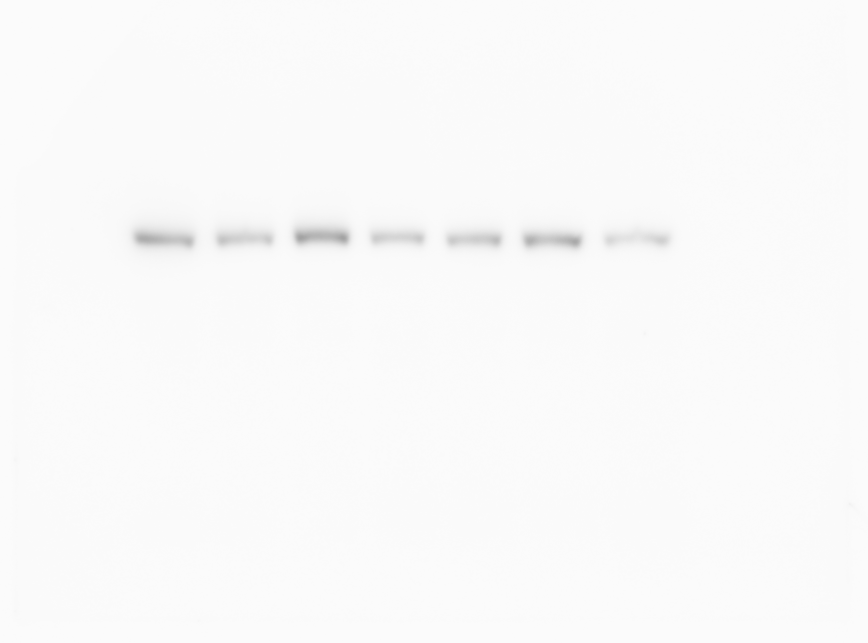


WT

WT

dKO

dKO

dKO

dKO

WT

Akt total

**Figure 2: Ablation of LXR does not alter normal myelination and redox status during development.**

The images below are the full-length blots used for the quantification of Nrf2, MPZ and PMP22 in Figure 2.

All protein levels were normalized to alpha tubulin.


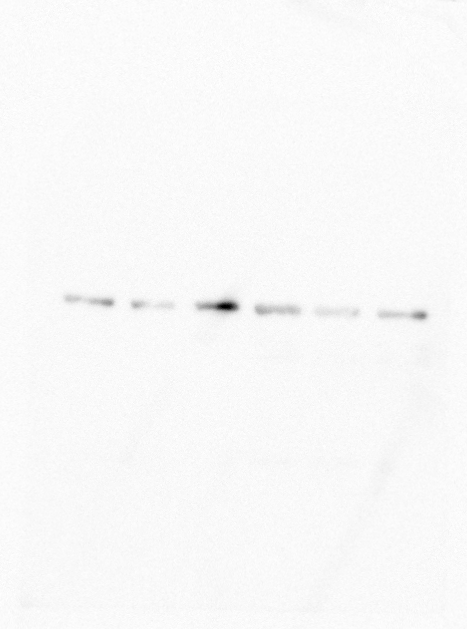

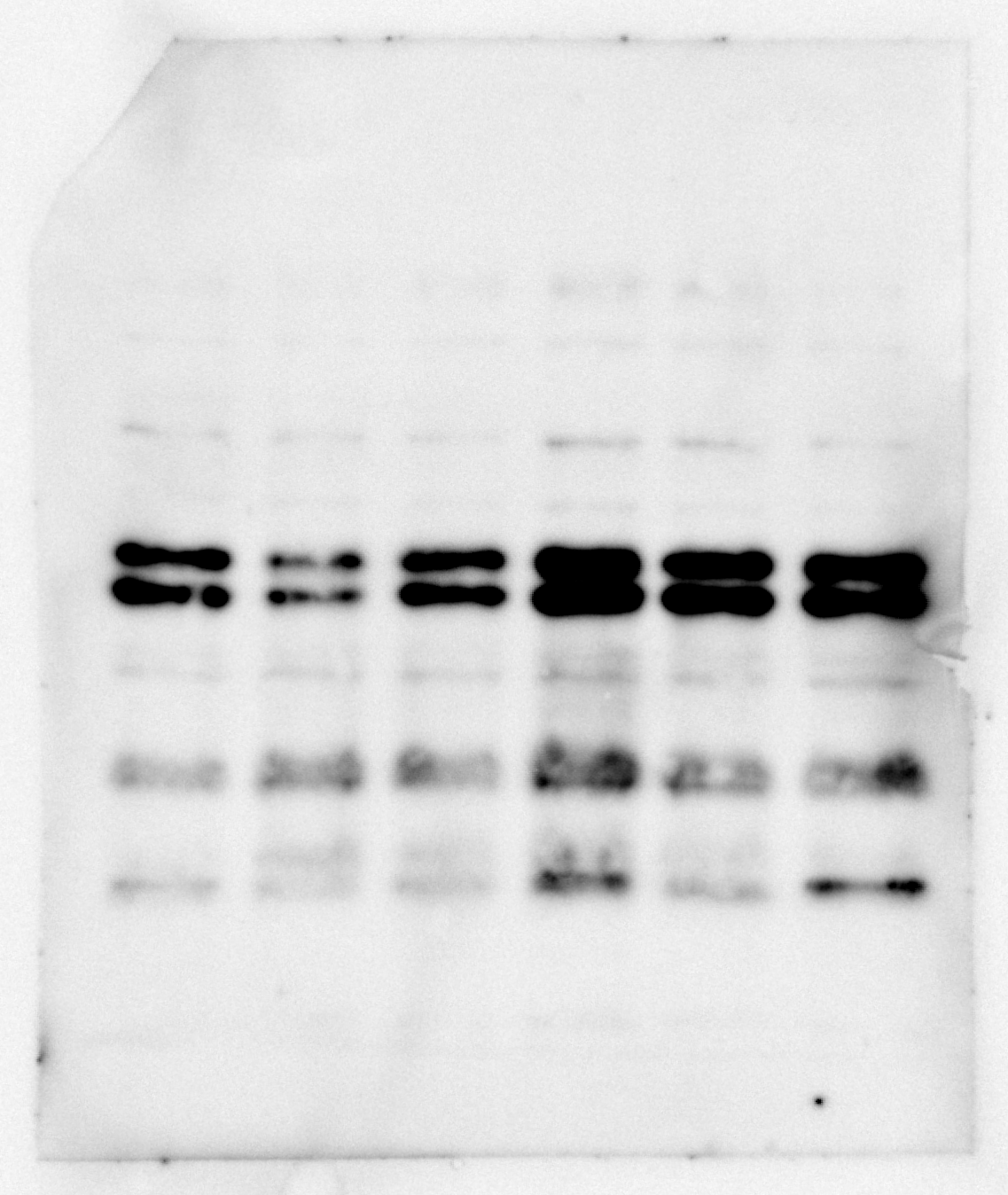


NRF2

a-tub

WT P21

dKO P21

WT P21

dKO P21


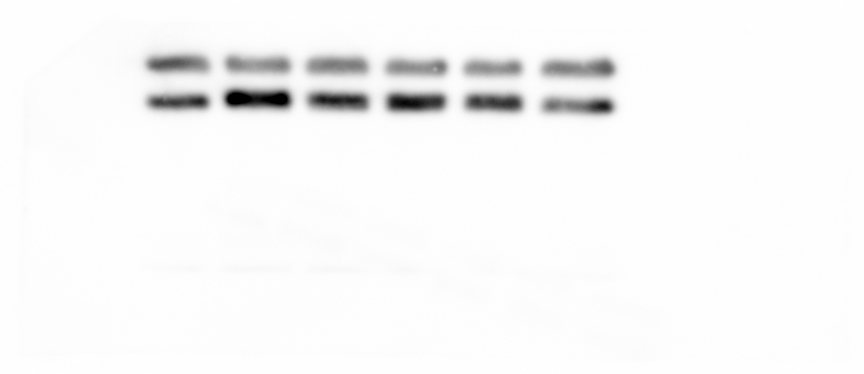


WT P21

dKO P21

MPZ


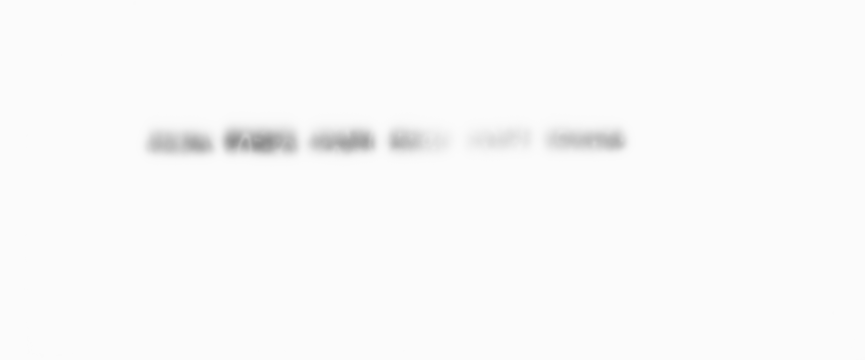


WT P21

dKO P21

PMP22

**Figure 3: N-acetylcysteine treatment markedly attenuates demyelination in LXRdKO mice**

The images below are the full-length blots used for the quantification of MPZ, PMP22

All protein levels were normalized to alpha tubulin.


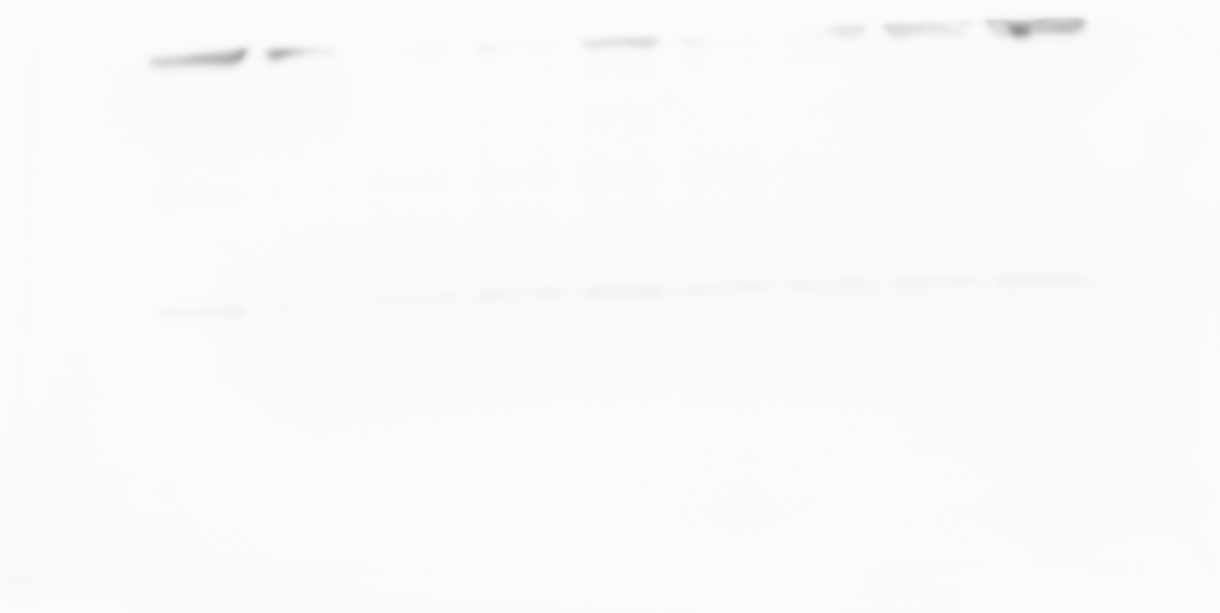


a-tub


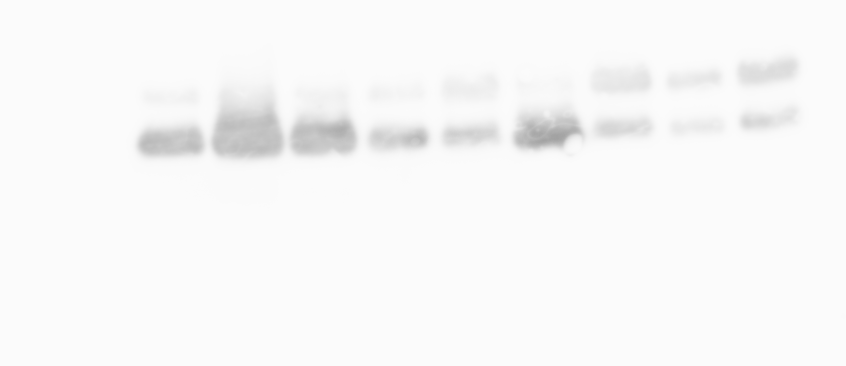


MPZ

WT

dKO

dKO +NAC


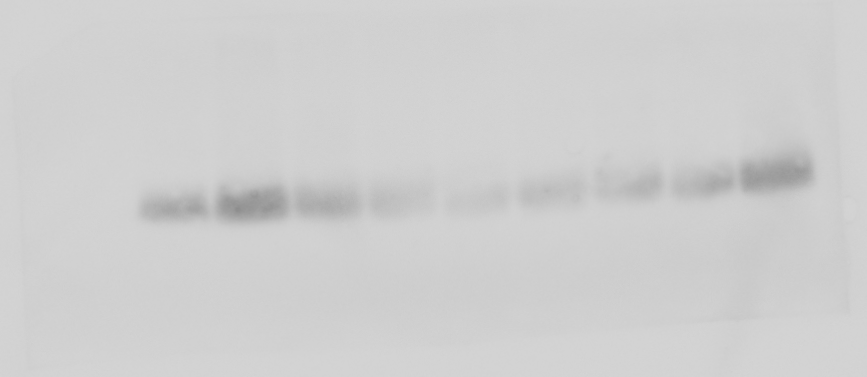


PMP22

WT

dKO

dKO +NAC

WT

dKO

dKO +NAC

**Figure 4: LXR activation by TO901317 regulates anti-oxidant enzyme expression in Schwann cells**

The images below are the full-length blots used for the quantification of Nrf2, Akt and P-Akt in Figure 4.


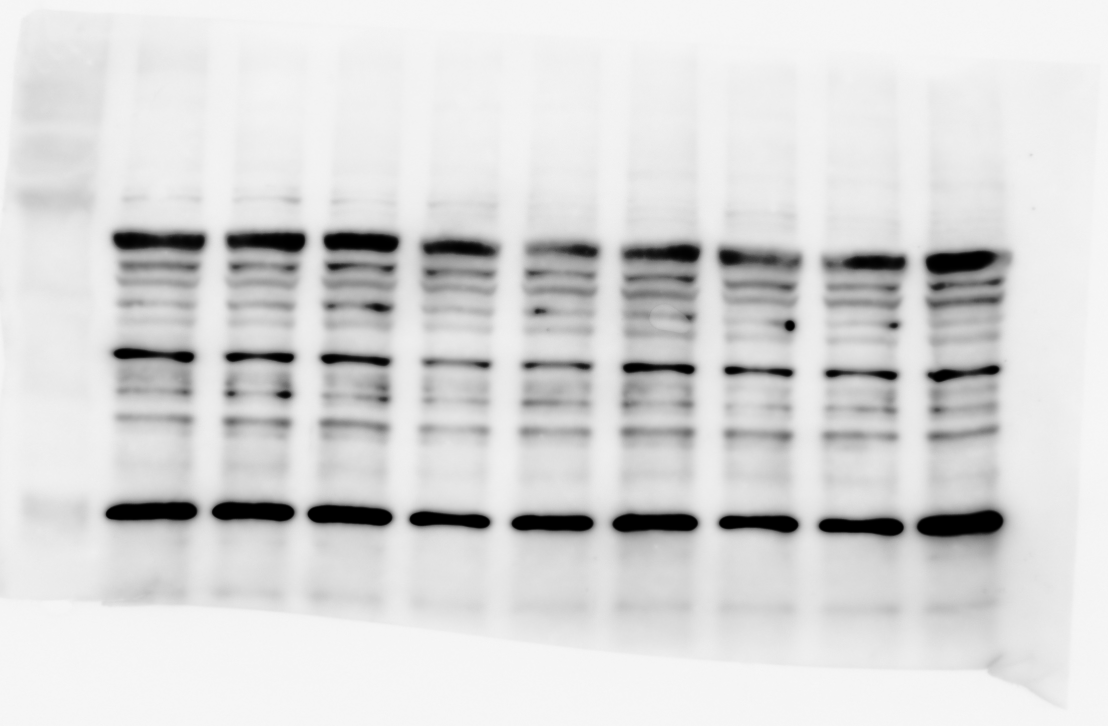


P-AKT

ETOH 2h

TO9 2h

ETOH 4h

TO9 4h

ETOH 8h

TO9 8h


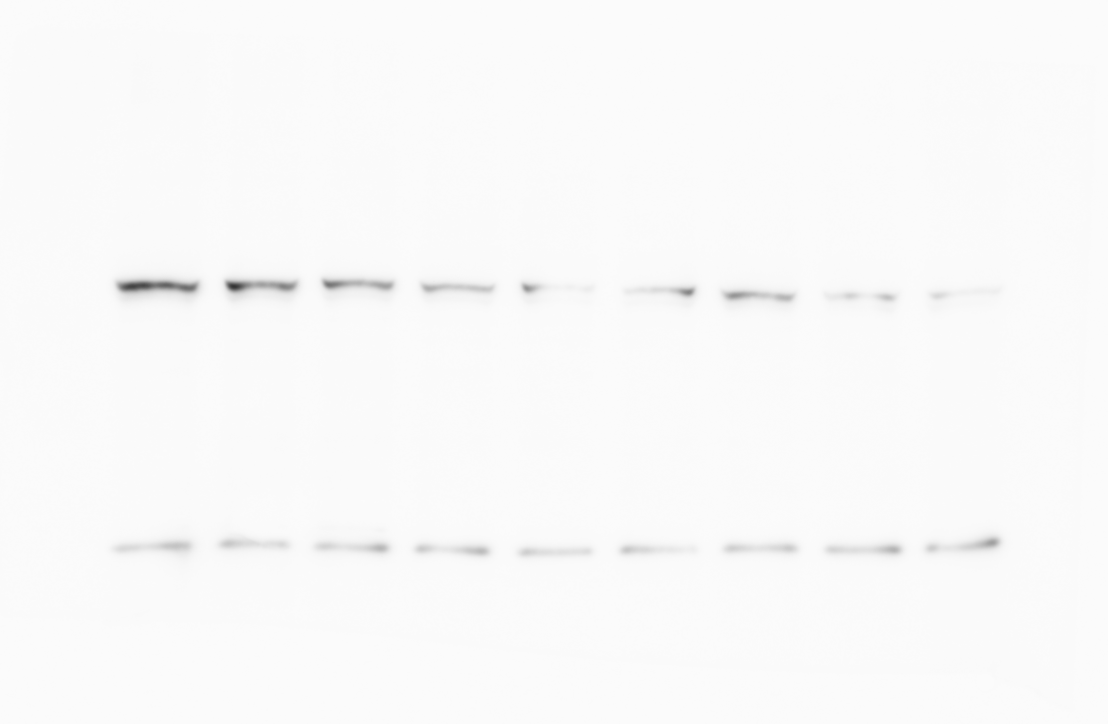


ETOH 2h

TO9 2h

ETOH 4h

TO9 4h

ETOH 8h

TO9 8h

AKT total
